# Rational Design of Monolithic g-C_3_N_4_ with Floating Network Porous-like Sponge Monolithic Structure for Boosting Photocatalytic Degradation of Tetracycline under Simulated and Natural Sunlight Illumination

**DOI:** 10.3390/molecules28103989

**Published:** 2023-05-09

**Authors:** Delu Cao, Xueying Wang, Hefan Zhang, Daiqiong Yang, Ze Yin, Zhuo Liu, Changyu Lu, Feng Guo

**Affiliations:** 1 School of Water Resource and Environment, Hebei Province Key Laboratory of Sustained Utilization & Development of Water Recourse, Hebei Province Collaborative Innovation Center for Sustainable Utilization of Water Resources and Optimization of Industrial Structure, Hebei Geo University, Shijiazhuang 050031, China; caodelu7@163.com (D.C.); 18239692184@163.com (X.W.); zhang020518@163.com (H.Z.); yangdaiqiong0510@126.com (D.Y.); yinze90@126.com (Z.Y.); zhuozi800725@163.com (Z.L.); 2 School of Material Science and Engineering, Jiangsu University of Science and Technology, Zhenjiang 212003, China

**Keywords:** photocatalysis, g-C_3_N_4_, floating catalyst, natural sunlight, tetracycline

## Abstract

In order to solve the problems of powder g-C_3_N_4_ catalysts being difficult to recycle and prone to secondary pollution, floating network porous-like sponge monolithic structure g-C_3_N_4_ (FSCN) was prepared with a one-step thermal condensation method using melamine sponge, urea, and melamine as raw materials. The phase composition, morphology, size, and chemical elements of the FSCN were studied using XRD, SEM, XPS, and UV–visible spectrophotometry. Under simulated sunlight, the removal rate for 40 mg·L^−1^ tetracycline (TC) by FSCN reached 76%, which was 1.2 times that of powder g-C_3_N_4_. Under natural sunlight illumination, the TC removal rate of FSCN was 70.4%, which was only 5.6% lower than that of a xenon lamp. In addition, after three repeated uses, the removal rates of the FSCN and powder g-C_3_N_4_ samples decreased by 1.7% and 2.9%, respectively, indicating that FSCN had better stability and reusability. The excellent photocatalytic activity of FSCN benefits from its three-dimensional-network sponge-like structure and outstanding light absorption properties. Finally, a possible degradation mechanism for the FSCN photocatalyst was proposed. This photocatalyst can be used as a floating catalyst for the treatment of antibiotics and other types of water pollution, providing ideas for the photocatalytic degradation of pollutants in practical applications.

## 1. Introduction

As a typical antibiotic, tetracycline (TC) ranks second in production and usage globally and has been widely used in medicine and industry. However, TC has a complex structure and is a difficult-to-degrade organic pollutant that can easily accumulate in the environment. Moreover, TC has issues such as ecotoxicity and poor biodegradability, and residual TC in the environment may also increase microbial resistance, posing a serious threat to ecosystems and human health. Therefore, it is necessary to remove TC from the water environment [1,2,3]. The current mainstream methods for water purification include chemical air flotation, advanced oxidation, photocatalytic degradation, adsorption, and microbial treatment [4,5,6]. However, traditional treatment methods, such as chemical, physical, and biological methods, have some limitations, such as easily causing secondary pollution, incomplete degradation, and toxicity for microorganisms [7,8,9]. Photocatalytic technology is an advanced oxidation technology with low energy consumption, high reaction efficiency, and no secondary pollution [10,11,12,13]. Photocatalytic technology utilizes clean solar energy as the driving force to generate free radical active substances, such as superoxide radicals (·O_2_^−^) and hydroxyl radicals (·OH), by stimulating photo-generated carriers with strong redox ability in semiconductor materials [14,15,16], which thereby react with different pollutants and, ultimately, generate small molecules, such as CO_2_ and H_2_O, resulting in the removal of pollutants [17,18,19,20]. Therefore, photocatalytic technology provides a promising method for the removal of TC in aquatic environments.

Among the many photocatalytic materials, carbon nitride (g-C_3_N_4_) is favored by many researchers due to its advantages of being metal-free, easy to prepare, and low cost and having high chemical stability; it is thus widely used in research directions such as hydrogen production and pollutant degradation [21,22,23,24,25]. However, due to the limitations of the material properties, g-C_3_N_4_ powders are affected by defects such as easy aggregation, a low light response range, and a high photo-generated electron–hole recombination rate [26,27,28,29]. In addition, when added to polluted wastewater, g-C_3_N_4_ powders sink to the bottom of the water environment, making recovery difficult and resulting in the powders not being fully utilized [30,31,32]. Fortunately, preparing floating photocatalysts can address these issues, as they can fully utilize light energy and are easy to recover and reuse [33]. For example, a ZnFe_2_O_4_@SiO_2_@TiO_2_ composite floater prepared according to the sol–gel approach was used for dyestuff degradation by Meng et al. [34]. Under visible light conditions, the removal efficiency could reach 95.1% within 2 h. However, the abovementioned floating photocatalysts are rather complicated to prepare, so there is an urgent need to find a simple method for preparing floating photocatalysts [35]. The three-dimensional (3D) structure of melamine sponge (MS) gives it the advantages of high specific surface area, high nitrogen content, and high elasticity, making it an ideal carrier for preparing floating photocatalytic materials [36,37]. In addition, the carbonization of MS can form a carbon skeleton with a porous mesh structure, which provides sufficient attachment sites for the loading of other materials and effectively enhances the adsorption and catalytic performance of MS. For example, the ZIF-8/carbon-nitrogen foam synthesized by Daeok Kim et al. [38] was used for stratified pore oil capture and chemical fixation of CO_2_, and the ZIF-8/CN foam was found to reject water while exhibiting a very high affinity for oil.

In this study, g-C_3_N_4_ was successfully attached to the surface of MS using a one-step thermal shrinkage method, and an FSCN photocatalyst was prepared and applied to the photocatalytic degradation of TC. The floating network porous-like sponge monolithic structure of FSCN facilitates recycling and makes it possible to avoid the occurrence of secondary pollution. The effects of different dosages and reaction conditions on the degradation performance of FSCN were studied. In addition, the morphology, degradation, and optical properties of the material were studied through various characterization techniques, and the mechanism of FSCN’s photocatalytic degradation of TC was analyzed. The stability of FSCN was verified through cyclic experiments and tested, demonstrating the possibility of its practical application.

## 2. Results and Discussion

### 2.1. XRD Analysis

**Figure 1:**
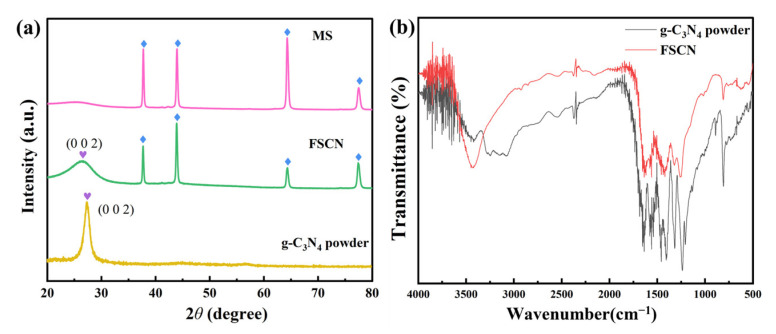
(**a**) XRD patterns for MS, FSCN, and g-C_3_N_4_ powders; (**b**) FT-IR patterns for FSCN and g-C_3_N_4_ powders.

Figure 1a shows the X-ray diffraction (XRD) results for the MS, FSCN, and g-C_3_N_4_ powders to illustrate each sample’s crystal structure and phase composition. It can be seen from the figure that the characteristic diffraction peaks of FSCN (37.5°, 43.9°, 64.3°, and 77.3°) [39] were similar to those of the MS, and the peak intensities had some subtle changes, which may have been due to the evolution of the morphology of melamine after calcination at high temperature. In accordance with g-C_3_N_4_ powders (JCPDS 87-1526) [40,41], the XRD pattern for FSCN showed a characteristic diffraction peak at 26.7° (002), attributed to the layer stacking of the in-planar repeating units of the continuous heptazine skeleton and the conjugated aromatic structure with a spacing of 0.32 nm [42,43]. The diffraction peak of FSCN was weaker, indicating that the interlayer period relevance length of the tri-s-triazine building block was reduced [44,45]. In summary, FSCN exhibited the characteristics of both MS and g-C_3_N_4_, proving that g-C_3_N_4_ successfully adhered to the MS with a network porous-like structure.

In order to verify the XRD results and further analyze the composition of FSCN, Fourier-transform infrared spectroscopy (FT-IR) was used to analyze the FSCN and g-C_3_N_4_ powders, as shown in Figure 1b. A comparison showed that the FSCN and g-C_3_N_4_ powders were basically the same. However, there were some differences in peak strength, which may have been due to the morphological evolution of melamine after high-temperature calcination of FSCN. There was a clear characteristic peak at 500 cm^−1^–1000 cm^−1^ found in both materials, which is a typical vibration peak of the tri-s-triazine ring of g-C_3_N_4_, once again proving the successful combination of g-C_3_N_4_ with MS [46]. Moreover, the FSCN and g-C_3_N_4_ powders exhibited a series of dense elastic vibration peaks in the range from 1000 cm^−1^ to 1700 cm^−1^, which can be attributed to the C-N and C=N vibration peaks of g-C_3_N_4_ [47]. The characteristic peaks of FSCN at 3000 cm^−1^–3500 cm^−1^ showed a certain deviation from the findings for the g-C_3_N_4_ powders, which may have been influenced by the MS. The characteristic peaks here can be attributed to the N-H and O-H of g-C_3_N_4_ [48]. The dense fluctuations at 3500 cm^−1^–4000 cm^−1^ may have been caused by potassium bromide doping during the testing process, which did not affect the analysis of the results [49]. In summary, FSCN exhibited all the characteristic peaks of the g-C_3_N_4_ material, indicating that g-C_3_N_4_ successfully combined with MS to form FSCN with the advantages of both materials, which was also consistent with the XRD results.

### 2.2. Morphology

**Figure 2:**
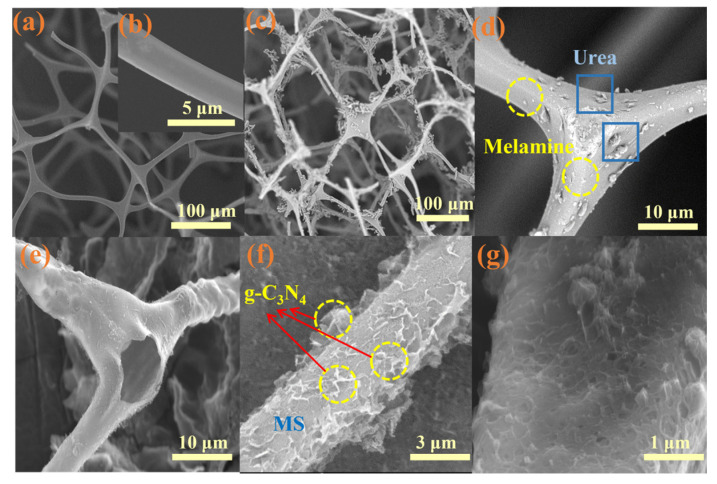
Scanning electron microscope results: (**a**,**b**) MS, (**c**,**d**) sponge soaked in urea and melamine, and (**e**–**g**) FSCN.

As shown in Figure 2a, MS exhibited a simple network of pore-like structures with a very high specific surface area. These structures provide the MS with abundant attachment points, making it an excellent carrier. Zooming-in to the 5 μm scale (Figure 2b), the MS can be seen as having a micron-rod structure with a diameter of 5 μm and a very smooth surface. After soaking with urea and melamine, the network porous-like structure of MS was not destroyed (Figure 2c), and it was also found that the surface of the MS became very rough, which further enhanced its material loading capacity, and urea and melamine particles were clearly visible on the MS (Figure 2d). After high-temperature calcination, it could be clearly seen that the FSCN had a hollow structure (Figure 2e), and the urea and melamine initially wrapped around the three-dimensional skeleton transformed into sheet-like g-C_3_N_4_, finally forming FSCN with a network porous-like structure, which was consistent with the XRD and FT-IR results. Moreover, the problem of the easy aggregation of the g-C_3_N_4_ monomer was solved. Additionally, as Figure 2f shows, the surface roughness of the material was further enhanced and, together with the flaky g-C_3_N_4_, this provided a greater specific surface area, which was more conducive to the adhesion of pollutants and improved the efficiency of contaminant treatment, providing a high reference value for the in-depth study of related materials.

### 2.3. XPS

**Figure 3:**
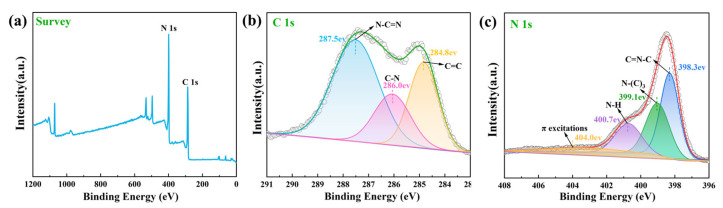
The XPS spectrum of the FSCN sample: (**a**) survey spectrum, (**b**) C1s, (**c**) N1s.

As shown in Figure 3, X-ray photoelectron spectroscopy (XPS) was used to observe the surface chemical composition and elemental valence states of the FSCN samples. Figure 3a shows the XPS survey spectrum for FSCN, and it is evident that the FSCN was mainly composed of C and N elements since the primary substance in FSCN was g-C_3_N_4_. Figure 3b shows the high-resolution spectrum for C1s. After fitting, the C1s peak of the FSCN sample could be divided into three peaks at 287.5 eV, 286.0 eV, and 284.8 eV, respectively. The characteristic peak at 287.5 eV was formed due to N-C=N, the presence of C-N resulted in a characteristic peak at 286.0 eV, and the characteristic peak at 284.8 eV can be attributed to the existence of C=C [50]. The high-resolution spectrum for N1s is shown in Figure 3c, and the N1s of FSCN was fit to four peaks, with the peaks located at 398.3 eV, 399.1 eV, and 400.7 eV corresponding to C=N-C, N-(C)_3_, and N-H. The peak at 404.0 eV can be attributed to the excitation of π electrons in the C=N conjugated structure [51,52].

### 2.4. UV–Visible Spectroscopy

**Figure 4:**
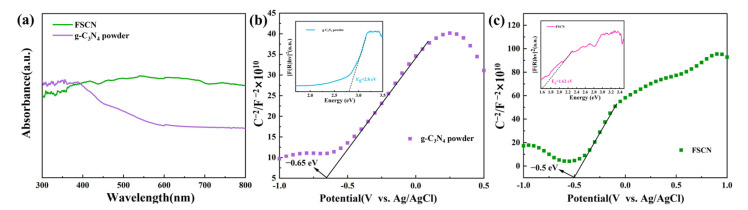
(**a**) UV–visible spectroscopy results for FSCN and g-C_3_N_4_ powders. Mott–Schottky test results and Eg values: (**b**) FSCN, (**c**) g-C_3_N_4_ powder.

UV–visible diffuse reflectance spectroscopy was used to determine the optical absorption range and energy band gap of the FSCN and g-C_3_N_4_ powder samples. As shown in Figure 4a, both the FSCN and g-C_3_N_4_ powders had strong absorption capabilities in the UV and visible light regions. FSCN had a black carbonized structure that was more conducive to light absorption and a porous mesh structure that could reflect incident light multiple times, thereby improving the light utilization efficiency. This indicated that the black sponge-like monolithic structure further improved the utilization of light by FSCN, thus enhancing the photocatalytic activity. The forbidden bandwidths for the FSCN and g-C_3_N_4_ powders were calculated using the Tauc plot equation, and the results are shown in Figure 4b,c. The band gaps of the FSCN and g-C_3_N_4_ powders were 1.62 eV and 2.8 eV, respectively. The narrower band gap of FSCN compared to that of g-C_3_N_4_ powder gives it a better ability to utilize visible light, thus improving the photocatalytic performance. Moreover, these findings strongly agree with the previous SEM and XRD characterization results. Subsequently, the flat-band (FB) potentials and semiconductor types of the FSCN and g-C_3_N_4_ powders were examined using electrochemical Mott–Schottky analysis [53]. The linear plots of both the FSCN and g-C_3_N_4_ powders had positive slopes, which indicated that both the FSCN and g-C_3_N_4_ powders were n-type semiconductors. Remarkably, the FB potential was 0.1 V higher than the conduction-band (CB) potential of the n-type semiconductor [54]. The FB potential of the g-C_3_N_4_ powder was −0.65 V (−0.45 V vs. NHE), and the FB potential of FSCN was −0.5 V (−0.3 V vs. NHE) according to the Nernst formula: E_NHE_ = E_Ag/AgCl_ + 0.197 [55]. Thus, the FSCN and g-C_3_N_4_ powders’ CBs were −0.4 eV and −0.55 eV, respectively. The VBs of the FSCN and g-C_3_N_4_ powders were 1.22 eV and 2.25 eV, respectively, according to the equation E_VB_ = E_g_ + E_CB_.

### 2.5. Photocatalytic Activities

**Figure 5:**
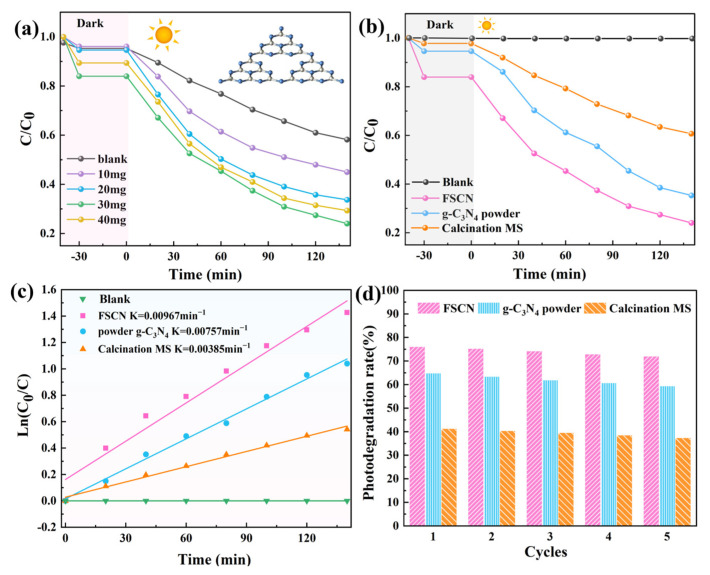
(**a**) Degradation of TC by FSCN; (**b**) degradation of TC by FSCN, g-C_3_N_4_ powder, and calcination MS; (**c**) first-order reaction kinetics of TC degradation with FSCN, g-C_3_N_4_ powder, and calcination MS; (**d**) cycle experiment results for FSCN, g-C_3_N_4_ powder, and calcination MS material.

Figure 5 shows the photocatalytic activity of FSCN, calcination MS, and g-C_3_N_4_ powder on TC under xenon lamp irradiation. Figure 5a shows the effects of different FSCN dosages on the performance of degraded TC (40 mg/L). The figure shows that TC did not self-degrade under xenon lamp irradiation without the catalyst. The removal rates for TC achieved with 10 mg, 20 mg, and 30 mg of FSCN were 55%, 66.3%, and 76%, respectively. This indicated that the degradation rate for TC continuously increased with the increase in FSCN dosage and finally reached equilibrium. However, when the FSCN dosage was 40 mg, the removal rate for TC decreased by 5.3% because too much of the photocatalyst led to shielding and scattering of light, thus limiting the photocatalytic activity of the material and reducing the photocatalytic efficiency. Therefore, the optimal catalyst dosage for FSCN was 30 mg. In addition, to make the data more convincing, the photocatalytic activities of FSCN, MS, and g-C_3_N_4_ powder were tested for comparison. As shown in Figure 5b, the TC concentration in all three groups of samples gradually decreased with the increase in light time. Among them, the highest TC removal rate achieved with FSCN reached 76%, which was 11.3% higher than that of the g-C_3_N_4_ powder (64.7%) and 34.8% higher than that of the MS (41.2%). It is apparent from Figure 5c that the photocatalytic degradation data for all three samples pertained to first-order reaction kinetics. The kinetic constants K of the products were obtained by calculation, and the K values for FSCN (0.00967 min^−1^) were 1.28 and 2.51 times higher than those for g-C_3_N_4_ powder (0.00757 min^−1^) and calcination MS (0.00385 min^−1^), respectively. In order to evaluate the photocatalytic stability of the FSCN, three cycle experiments were performed. As shown in Figure 5d, the photocatalytic degradation of TC by FSCN, calcination MS, and g-C_3_N_4_ powder decreased by 1.9%, 1.7%, and 2.9%, respectively, after three cycle reactions, which indicated that the material was more stable after the addition of MS. In summary, the FSCN prepared by calcination with MS as the carrier showed an increased specific surface area and provided more active sites due to the sponge’s monolithic three-dimensional-network porous structure, thus effectively promoting the adsorption of TC and improving the photocatalytic degradation rate and photocatalytic activity. Moreover, compared with g-C_3_N_4_ powder, FSCN had a higher recycling rate and better photostability.

**Figure 6:**
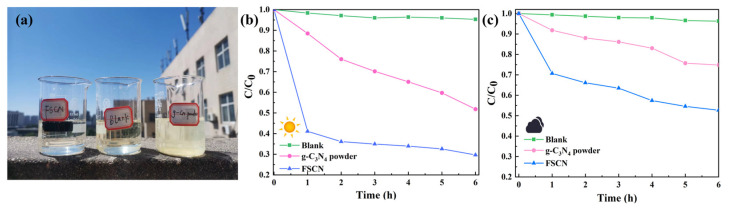
(**a**) Photocatalytic removal of TC under natural light conditions. Photocatalytic removal performance of FSCN and g-C_3_N_4_ powder with TC: (**b**) sunny day (**c**) cloudy day.

In order to simulate a practical application scenario, the photocatalytic activities of the FSCN and g-C_3_N_4_ powder on TC were tested under natural sunlight illumination. As shown in Figure 6a, the g-C_3_N_4_ powder was all deposited in the water. At the same time, the FSCN with a porous network structure could float on the water surface during the photocatalytic reaction, making it easy to recycle. Moreover, a set of blank experiments were set up as a control group to ensure the accuracy of the experimental results. Two sets of experiments were set up under sunny and cloudy conditions to investigate the effect of weather on the photocatalytic performance of the materials. First, in the reaction under sunny conditions, after 6 h, FSCN could remove 70.4% of the TC. The degradation of TC by g-C_3_N_4_ powder was only 48.3% (Figure 6b). The photocatalytic performances of the FSCN and g-C_3_N_4_ powders were significantly inhibited under cloudy conditions, with removal rates of 47.3% and 25.2%, respectively (Figure 6c). As mentioned above, FSCN maintains excellent photocatalytic activity under sunlight and can be recycled without secondary pollution.

### 2.6. Electrochemical Test

**Figure 7:**
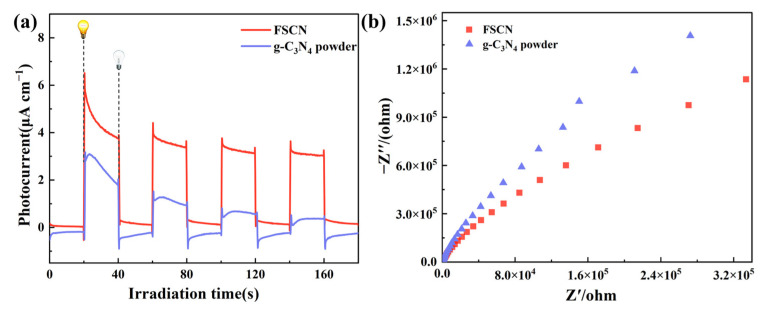
FSCN and g-C_3_N_4_ powder: (**a**) transient photocurrent diagram; (**b**) electrochemical impedance spectrum.

The electron–hole migration and separation efficiency of the FSCN and g-C_3_N_4_ powder samples were analyzed using the photocurrent response and electrochemical impedance under visible light. Figure 7a shows the instantaneous photocurrent responses of the FSCN and g-C_3_N_4_ powder samples. The current density of the g-C_3_N_4_ powder was significantly lower than that of FSCN, indicating that calcinating FSCN with MS as a carrier could effectively improve the photogenerated carrier separation efficiency of the material compared to g-C_3_N_4_ powder. Figure 7b displays the AC impedance spectra for FSCN and the g-C_3_N_4_ powder. The radius of curvature of FSCN was smaller than that of g-C_3_N_4_, indicating that the resistance of FSCN was lower than that of the g-C_3_N_4_ powder, resulting in better conductivity, more significant separation efficiency for photogenerated carriers, and superior photocatalytic performance.

### 2.7. Radical Trapping and ESR

**Figure 8:**
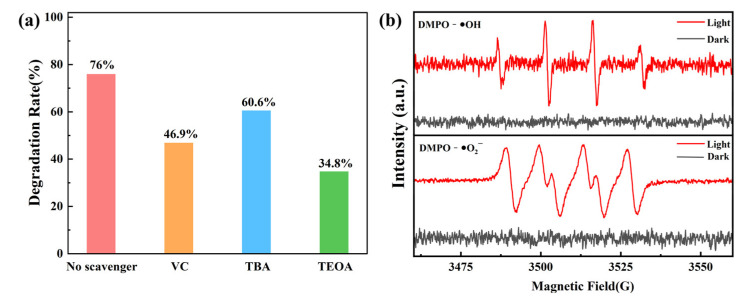
(**a**) Active-species trapping experiments for visible photocatalytic degradation of TC by FSCN and (**b**) ESR spectra of FSCN under visible light and dark conditions.

Reactive radical trapping experiments and electron spin resonance (ESR) spectroscopy were used to verify the photocatalytic mechanism and explore the role of each reactive species during the reaction. VC, TBA, and TOEA were selected as trapping agents and added to the reaction system to trap ∙O_2_^−^, ∙OH, and h^+^, respectively, and determine their contributions to the reaction. As shown in Figure 8a, the rate of degradation of TC decreased from 76% to 46.9% after the addition of ∙O_2_^−^, which indicated that ∙O_2_^−^ played a more important role in the reaction process. The rate of degradation of TC was most obviously inhibited after the addition of TOEA, decreasing to 34.8%, indicating that h^+^ was the most critical active factor in the degradation process. In contrast, the simple degradation rate for TC was only reduced by 15.4% after the addition of TBA, which indicated that ∙OH was involved in the reaction but its contribution was low. Notably, the production of ∙OH was inhibited in the process of h^+^ capture, further indicating the important role of h^+^ in the degradation process. To further verify the effects of the active factors on degradation, ESR analysis was performed under visible light. Figure 8b shows that no EPR signals for DMPO-∙O_2_^−^ or DMPO-∙OH were observed under dark conditions. However, a series of distinctive characteristic peaks appeared under light conditions, which indicated that ∙O_2_^−^ and ∙OH radicals were involved in the photocatalytic reaction and played key roles.

### 2.8. Photocatalytic Mechanism

**Figure 9:**
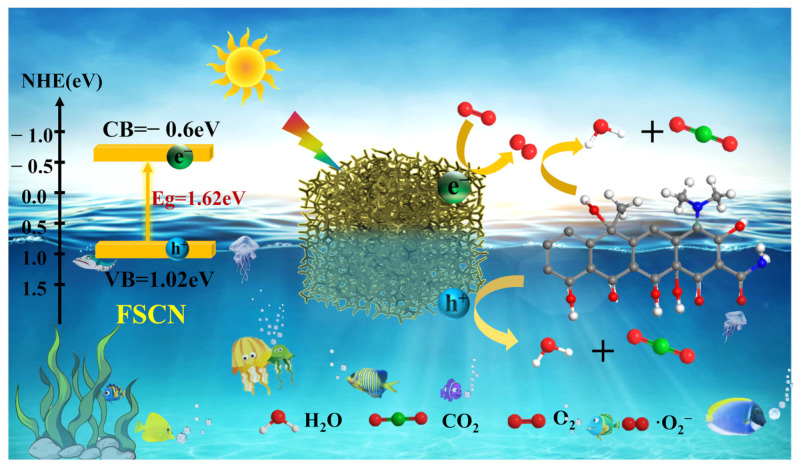
Diagram of the mechanism of TC degradation by FSCN photocatalytic material under visible light.

Based on the above experiments and analysis, a possible photocatalytic reaction mechanism for the degradation of TC by FSCN under visible light irradiation can be proposed. As shown in Figure 9, under the irradiation of visible light, the electron (e^−^) on the valence band (VB) of the FSCN leapfrogged above the conduction band (CB), leaving the hole (h^+^) on the VB. Since the CB potential of FSCN was more negative at −0.4 eV compared to −0.33 eV, the e^−^ clustered in the CB could react with O_2_ to form superoxide radicals (∙O_2_^−^) with strong oxidation properties. Meanwhile, the potential on the VB of the FSCN was 1.22 eV, significantly lower than 1.99 eV, so the h^+^ on the VB was insufficient to react with H_2_O and OH^−^ to form hydroxyl radicals (∙OH). In summary, in the FSCN photocatalytic system, the main active species that react with TC in wastewater are ∙O_2_^−^ and h^+^. The TC undergoes redox reactions with mineralization to produce small molecules, such as CO_2_ and H_2_O, which are eventually removed in the water.

## 3. Materials and Methods

### 3.1. Chemicals and Materials

Both urea and melamine were purchased from Chengdu Aikeda Chemical Reagent Co. (Chengdu, China). Melamine sponge was supplied by Tianjin Damao Chemical Reagent Factory (Tianjin, China), and tetracycline was purchased from Shanghai Baoman Biotechnology Co. (Shanghai, China). All experimental water was pure water, and all chemical substances used in the experiment were analytical grade and used without further purification.

### 3.2. Characterization

A D/MAX-2500VL/PC (Rigaku Co., Tokyo, Japan) was used for the X-ray diffraction (XRD) analysis of FSCN, and powder g-C_3_N_4_ photocatalysts were analyzed in the range from 20 to 80 in the 2θ. A S-4800 scanning electron microscope (SEM) (Hitachi Co., Tokyo, Japan) was used to characterize and analyze the morphology and size of the FSCN and powder g-C_3_N_4_ photocatalysts. An ESCALAB250Xi X-ray photoelectron spectrometer (XPS) (Thermo Co., Waltham, MA, USA) was used to characterize the chemical state and composition of the photocatalysts. A UV-2550 was used for diffuse reflectance spectroscopy (UV–visible spectroscopy) to analyze the optical absorption properties of the photocatalytic materials. A CHI660D electrochemical workstation (Chenhua Co., Ltd., Shanghai, China) was used to study the separation of the photogenerated carriers of the catalyst.

### 3.3. Synthesis of g-C_3_N_4_ Powder and FSCN

For the next step, 6 g of urea and 8 g of melamine were mixed thoroughly and added to a semi-open corundum crucible. The mixture was placed in a tube furnace and heated to 550 °C at a heating rate of 5 °C/min in a N_2_ environment and then calcined at a constant temperature for 4 h. After the reaction, the light yellow product was cooled to room temperature and ground to obtain g-C_3_N_4_ powder.

**Figure 10:**
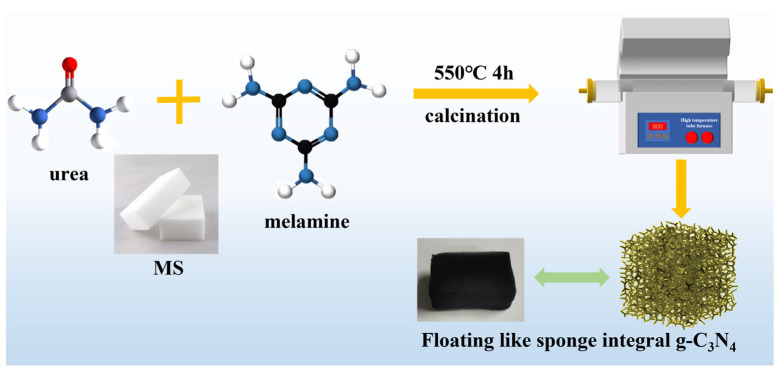
Diagram of the preparation of FSCN photocatalytic materials.

Then, 16 g of urea was weighed and dissolved into 40 mL of deionized water, after which 8 g of melamine was added and the mixture was stirred magnetically for 3 h at room temperature to obtain a saturated solution. The MS was cut to obtain a rectangle of 4 × 2 × 2 cm^3^, and its mass was recorded. The MS was immersed in the above solution. After that, the MS was taken out and dried in a freeze dryer for 24 h and then placed in a corundum crucible and heated to 550 °C in a tube furnace with a 5 °C/min heating rate in a N_2_ environment for 4 h of calcination. After cooling to room temperature, the synthesized product was washed with deionized water and ethanol alternately three times. Then, the samples were dried in a vacuum oven at 80 °C for 10 h and cooled naturally to obtain 2.2 × 1.5 × 1.5 cm^3^ of black floating network porous-like sponge monolithic structure g-C_3_N_4_, which was named FSCN. The preparation principle is shown in Figure 10.

### 3.4. Measurement of Photocatalytic Activity

#### 3.4.1. Indoor Experiment

First, 30 mg of FSCN, g-C_3_N_4_ powder, and calcined MS were respectively weighed and added to 50 mL quartz test tubes. Afterward, 40 mg/L TC solution was added to the quartz test tubes as the target pollutant and a set of quartz test tubes with only TC solution were used as a blank control. Next, the quartz test tubes were placed in a photocatalytic reactor and stirred with magnetic force in a dark environment for 30 min to achieve a dark reaction and ensure that the FSCN, pure calcined sponge, and powdered g-C_3_N_4_ photocatalysts achieved adsorption–desorption equilibrium for TC and to reduce experimental errors. A 300 W xenon lamp was turned on as the light source for the photocatalytic reaction. Then, 3 mL of the solution was removed every 20 min and centrifuged for 5 min. After centrifugation, the supernatant was extracted. The wavelength of the UV–visible spectrophotometer was set to 357 nm, the absorbance of the supernatant was measured and recorded, and, ultimately, the degradation rates for TC achieved by the three photocatalysts were determined. The different masses of the FSCN were weighed, keeping the other conditions unchanged, and photocatalytic experiments were conducted to test the effect of dosage on photocatalytic performance.

#### 3.4.2. Outdoor Experiment

First, 60 mg of FSCN and 60 mg of g-C_3_N_4_ powder were weighed and poured into beakers filled with 100 mL of TC solution (40 mg/L). Next, a 30 min dark reaction was conducted to ensure adsorption equilibrium. Then, the samples were transferred to a natural sunlight-irradiated environment with a light intensity of 58.28 mW/cm^2^ for the photocatalytic reaction. The blank control group comprised 100 mL of 40 mg/L TC without a catalyst. In the next step, 3 mL of the solution was removed every hour and centrifuged, the supernatant was extracted, and the TC absorbance was measured and recorded with an ultraviolet–visible spectrophotometer at a wavelength of 357 nm. In addition, the sunny natural environment was replaced with a cloudy natural environment, with other experimental conditions kept the same as above, and the TC absorbance was measured and recorded.

### 3.5. Reactive Radical Trapping Experiments

Similar to the above photocatalysis experiment, the original experimental process was kept unchanged. Before the reaction, 1 mmol vitamin C (VC), thiobarbituric acid (TBA), and triethanolamine (TEOA) captors were added to the TC solution to explore the contributions of superoxide anion radicals (O_2_^−^), hydroxyl radicals (OH), and holes (h^+^) in the experiment on the process of photocatalysis.

## 4. Conclusions

This study successfully prepared g-C_3_N_4_ (FSCN) photocatalysts with a floating network porous-like sponge monolithic structure through a one-step thermal shrinkage method. The experimental results indicated that FSCN exhibited excellent photocatalytic performance and stability for TC degradation with both laboratory light sources and sunlight exposure. After a series of characterization analyses, it can be concluded that the factors affecting the photocatalytic performance of FSCN are the following: (1) the addition of MS effectively solved the problem of easy aggregation of g-C_3_N_4_, and the increase in the specific surface area provided more attachment sites for TC; (2) the black structure after MS carbonization was more conducive to light absorption, and the complex porous structure increased the refraction of light in the material and improved light utilization efficiency, thus improving photocatalytic performance. In addition, FSCN also has the advantages of being low cost and easy to prepare, allowing easy recovery, and producing no secondary pollution. This study provides new ideas for the design and preparation of recyclable photocatalysts that can help to reduce the harm from antibiotic pollution on the ecological environment and human health.

## Data Availability

The data from the study can be provided by the corresponding author upon reasonable request.

## Sample Availability

Samples of the compounds are available from the authors.
